# *Wisteria floribunda* agglutinin-sialylated mucin core polypeptide 1 is a sensitive biomarker for biliary tract carcinoma and intrahepatic cholangiocarcinoma: a multicenter study

**DOI:** 10.1007/s00535-016-1230-0

**Published:** 2016-06-29

**Authors:** Junichi Shoda, Atsushi Matsuda, Takashi Shida, Masakazu Yamamoto, Masato Nagino, Toshio Tsuyuguchi, Takahiro Yasaka, Susumu Tazuma, Kazuhisa Uchiyama, Michiaki Unno, Nobuaki Ohkohchi, Yasuni Nakanuma, Atsushi Kuno, Hisashi Narimatsu

**Affiliations:** 1 Department of Medical Science, Faculty of Medicine,, University of Tsukuba, 1-1-1 Tennodai, Tsukuba, Ibaraki 305-8575 Japan; 2 Research Center for Medical Glycoscience (RCMG), National Institute of Advanced Industrial Science and Technology (AIST), Central 2, Tsukuba, Ibaraki Japan; 3 Department of Surgery, Institute of Gastroenterology, Tokyo Women’s Medical University, Tokyo, Japan; 4 Division of Surgical Oncology, Department of Surgery, Nagoya University Graduate School of Medicine, Nagoya, Japan; 5 Department of Gastroenterology and Nephrology, Graduate School of Medicine, Chiba University, Chiba, Japan; 6 Division of Surgery, Nagasaki Prefectural Kamigoto Hospital, Nagasaki, Japan; 7 Department of General Internal Medicine, Hiroshima University Hospital, Hiroshima, Japan; 8 Department of General and Gastroenterological Surgery, Osaka Medical College, Takatsuki, Osaka Japan; 9 Department of Surgery, Tohoku University School of Medicine, Sendai, Japan; 10 Department of Gastrointestinal Surgery, Faculty of Medicine, University of Tsukuba, Tsukuba, Ibaraki Japan; 11 Department of Human Pathology, Graduate School of Medical Science, Kanazawa University, Kanazawa, Japan

**Keywords:** Biliary tract carcinoma, Intrahepatic cholangiocarcinoma, Biomarker, Glycoproteomics, Multicenter study

## Abstract

**Background:**

*Wisteria floribunda* agglutinin (WFA)-sialylated mucin core polypeptide 1 (MUC1) was investigated as a new glycoprotein marker for cholangiocarcinoma (CC) using glycoproteomics technologies. In this multicenter study, WFA-sialylated MUC1 levels in serum and bile samples were measured to determine their diagnostic capability in biliary tract carcinoma (BTC) and intrahepatic (Ih) CC.

**Methods:**

The study included 244 patients with BTC, 59 patients with IhCC, 287 patients with benign biliary tract diseases, and 44 control subjects.

**Results:**

Serum WFA-sialylated MUC1 levels were significantly higher in patients with either BTC or IhCC than in control subjects and those with benign biliary tract diseases. Patients with IhCC showed higher WFA-sialylated MUC1 levels than patients with tumors at other sites. No significant differences in WFA-sialylated MUC1 levels were found with regard to cancer stage or tissue type. Receiver operating characteristic curve analysis showed that WFA-sialylated MUC1 was superior to carbohydrate antigen 19-9 (CA19-9) and carcinoembryonic antigen (CEA) for the diagnosis of benign biliary tract diseases, BTC, and IhCC, as well as for stage I and II carcinomas. Significantly higher levels of biliary WFA-sialylated MUC1 were observed in BTC/IhCC than in benign biliary tract diseases. The diagnostic capability of biliary WFA-sialylated MUC1 was also superior to that of CA19-9, and diagnostic sensitivity was higher than that of biliary cytology for BTC/IhCC.

**Conclusions:**

WFA-sialylated MUC1 is a useful novel biomarker for BTC/IhCC. In the future, this measurement should be applied in the clinical setting.

## Introduction

Both biliary tract carcinoma (BTC) and intrahepatic (Ih) cholangiocarcinoma (CC) are associated with a poor overall prognosis. Although the worldwide incidence of these carcinomas is relatively low, the incidence of BTC and BTC-related mortality is high in Japan, Central and South America, and Eastern Europe [1, 2]. In Japan, the number of BTC-related deaths has risen to nearly 20,000 each year [3]. IhCC, in particular, is associated with high rates of morbidity and mortality globally [4–6].

BTC and IhCC have demonstrated unfavorable survival outcomes, which may be attributed to the complexity of the anatomical environment at sites of tumor occurrence and to the diverse modes of tumor progression—infiltration, dissemination, and metastasis. Moreover, because early detection of these types of cancer is difficult, even with a complete diagnostic workup and advanced imaging technology, surgical resection at the early stage is not feasible in most cases. Carcinogenesis and the mechanism of progression in BTC/IhCC may involve chronic persistent inflammation in the biliary epithelium, which is supported by the observation that the frequency of CC is higher in patients with primary sclerosing cholangitis [7], clonorchis sinensis [8], and hepatolithiasis [9]. Existing tumor markers have poor diagnostic utility because of the influence of co-existing inflammatory conditions of the biliary tract. Therefore, in order to improve prognosis for patients with BTC/IhCC, novel biomarkers for early diagnosis are urgently needed.

Glycans, often referred to as “the face of the cell,” and the glycan structure of mucin glycoprotein that forms the epithelial cell surface are altered by oncogenic transformation. Oncogenic transformation is associated with differences in expression profiles of glycogenes between normal cells and carcinoma cells. Most existing tumor markers are carbohydrate antigens. Glycans bound to the core protein are closely linked with biotics for carcinogenesis and cancer progression. Analysis of clinical samples from patients with CC has shown that abnormal mucin glycoproteins [10] and abnormal expression of glycosyltransferase [11, 12] are present in the tumor cells, and this has been found to have a significant impact on the malignant behaviors of the tumor.

Kuno and colleagues developed a glycan profiling system and method for glycoproteomics-based glycoprotein marker identification [13] that enables highly sensitive glycan analysis with a lectin microarray from the minimal domain of formalin-fixed clinical samples [14]. Comparative glycan profiling analysis showed that *Wisteria floribunda* agglutinin (WFA) was a useful lectin probe found in CC tissues [15]. In addition, this glycoproteomics-based approach with immunohistochemistry identified sialylated mucin core polypeptide 1 (MUC1), recognized with the MY.1E12 monoclonal antibody (mAb) [16], as a mucin glycoprotein molecule [15].

An analysis of multiple clinical samples requires a simplified measurement system. Matsuda and colleagues recently constructed a sandwich enzyme-linked immunosorbent assay (ELISA) with solid-phase WFA and MY.1E12 mAb overlaid [17]. Therefore, using this system, we conducted the current multicenter clinical study under the National Survey for Intractable Hepatobiliary Diseases by the Japanese Ministry of Health, Labour and Welfare (MHLW) to prospectively collect clinical samples from patients with either BTC or IhCC and to determine the levels of WFA-sialylated MUC1 in serum and bile. To study the clinical significance of WFA-sialylated MUC1, we compared levels in samples among different primary tumor sites, cancer stages, and tissue types. We also compared the diagnostic capability of WFA-sialylated MUC1 with that of conventional tumor markers and biliary cytology.

## Patients and methods

### Samples

This prospective clinical trial was organized by the study group for the National Survey for Intractable Hepatobiliary Diseases under the MHLW in Japan (Director, Dr. Yasuni Nakanuma), and was conducted from 2012 to 2014 at multiple institutions. The study group included the University of Tsukuba (Ibaraki, Japan), Tokyo Women’s Medical University (Tokyo, Japan), Nagoya University (Nagoya, Japan), Kamigoto Hospital (Nagasaki, Japan), Chiba University (Chiba, Japan), Hiroshima University (Hiroshima, Japan), Osaka Medical College (Osaka, Japan), Tohoku University (Miyagi, Japan), and the National Institute of Advanced Industrial Science and Technology (Ibaraki, Japan). The study protocol was approved by the official committee of the National Survey for Intractable Hepatobiliary Diseases. The study procedures were consistent with the ethical standards of the Declaration of Helsinki. Informed consent was obtained from each patient. A total of 303 consecutive patients with BTC or IhCC and 287 patients with benign biliary tract diseases from the study group, as well as 44 control subjects (without any hepatobiliary diseases) recruited from the University of Tsukuba Hospital, were enrolled in the study.

The sex, age, and clinicopathological features of the patients with BTC/IhCC, including preoperative serum levels of total bilirubin (T-Bil), aspartate aminotransferase (AST), alanine aminotransferase (ALT), γ-glutamyl transpeptidase (γ-GT), CA19-9, and carcinoembryonic antigen (CEA), are summarized in Table 1. In patients who underwent surgery for BTC/IhCC, the pathological features of tissue samples were assessed according to the TNM Classification of Malignant Tumours, 7th Edition [18]. Among the 303 patients with BTC or IhCC, the diagnoses were as follows: 244 BTC (117 perihilar CC, 71 distal CC, and 56 gallbladder carcinoma) and 59 IhCC. The diagnoses of 287 benign biliary tract disease cases included cholelithiasis, choledocholithiasis, hepatolithiasis, primary sclerosing cholangitis, and pancreaticobiliary maljunction.

Serum samples were collected from all patients in the study . Bile samples were collected from 183 consecutive patients with BTC/IhCC (95 perihilar CC, 50 distal CC, 28 gallbladder carcinoma, and 10 IhCC) and 115 patients with benign biliary tract diseases who underwent endoscopic naso-biliary drainage, percutaneous transhepatic biliary drainage, or endoscopic retrograde cholangiography. In patients with biliary obstruction, serum and bile samples were generally collected after the decompression of biliary dilatation. For biliary cytology, bile samples were centrifuged within 30 min, and a smear of the cell suspension was stained using a standard Papanicolaou technique, followed by imaging with a light microscope. Two experienced cytologists classified all cytology slides as positive (class V), suggestive of malignancy (class IIIb or IV), or negative (class I, II, or IIIa). Although sampling was performed multiple times in some patients, the results of the first samples were adopted.

**Table 1:** Baseline characteristics, WFA-sialylated MUC1 and other marker levels in the serum samples of the study patients

Characteristics	Control (*n* = 44)	Benign biliary disease (*n* = 287)	Total (*n* = 303)	Perihilar CC (*n* = 117)	Distal CC (*n* = 71)	Gallbladder carcinoma (*n* = 56)	Intrahepatic CC (*n* = 59)
Age (years)	49 (20–82)	68 (19–92)^a^	71 (33–101)^a^	71 (40–87)^a^	73 (40–95)^a,b^	69 (33–92)^a^	71 (36–101)^a^
Gender (male/female)	23/21	153/134	193/111	74/43	60/11	29/27	30/30
pStage (I/II/III /IV)	–	–	23/50/78/153	7/20/25/65	8/17/32/14	6/9/9/32	2/4/12/42
Histology (Pap/Well/Mod/Por)	–	–	25/79/157/43	8/29/63/17	7/22/30/12	7/16/25/8	3/12/39/6
T-Bil (mg/dl)	0.7 (0.4–3.9)	0.7 (0.3–15.7)	0.8 (0.2–20.5)	0.9 (0.4-20.5)	0.9 (0.3–19.9)	0.9 (0.2–9.6)	0.7 (0.2–14.0)
AST (U/L)	20 (11–124)	26 (12–824)	34 (11–1436)	37 (14-254)	35 (12–1436)^a^	26 (11–351)	31 (12–245)
ALT (U/L)	17 (6-101)	21 (5–767)	40 (6–552)^a^	46 (7–358)	44 (6–552)^a,b^	27 (10–488)	29 (7–287)
γGT (IU/L)	22 (9–417)	41 (8–1737)	155 (9–1596)^a,b^	202 (19–1477)^a,b^	201 (9–1568)^a,b^	67 (11–671)	134 (10–1596)^a^
WFA-sialylated MUC1 (µL/mL)	84 (0.6–230)	124 (25–594)	340 (56–2000)^a,b^	346 (131–1910)^a,b^	252 (121–804)^a,b^	325 (56–2000)^a,b^	498 (103–2000)^a,b^
CA19-9 (U/mL)	9 (0.3–85)	12 (0.2–1069)	74 (0.6–1314)^a,b^	83 (1.4–998)^a,b^	49 (0.6–1314)	48 (0.8–1033)^a,b^	152 (0.8–1118)^a,b^
CEA (ng/mL)	1.3 (0.6–5.1)	2.6 (0.2–16.6)	2.6 (0.3–92.4)^b^	2.4 (0.5–77.8)	2.6 (0.3–15.4)	2.4 (0.4–41.2)	3.2 (0.4–92.4)^a,b^

Values are expressed as medians (range)

Total represents the sum of cases with intrahepatic CC, perihilar CC, distal CC, and gallbladder carcinoma

*CC* cholangiocarcinoma, *Pap* papillary carcinoma, *Well* well-differentiated carcinoma, *Mod* moderately differentiated carcinoma, *Por* poorly differentiated carcinoma, *T-Bil* total bilirubin, *AST* aspartate aminotransferase, *ALT* alanine aminotransferase, *γGT* γ-glutamyl transpeptidase, *WFA* wisteria floribunda agglutinin, *MUC1* mucin core polypeptide 1, *CA19-9* carbohydrate antigen 19-9, *CEA* carcinoembryonic antigen

^a^ significantly different from control, control subject

^b^ different from benign biliary disease

### Sandwich ELISA for measurement of WFA-sialylated MUC1

The measurement of WFA-sialylated MUC1 levels was performed in a blinded fashion for malignant and benign diseases. WFA-immobilized MY.1E12 sandwich ELISAs were performed as described previously [15, 17]. All experiments were carried out in triplicate, and the mean value was used as the final value for each sample. A standard curve of WFA-sialylated MUC1 was created using the media of TGBC-1-TKB human gallbladder cancer cells [19] provided by Dr. T. Todoroki (University of Tsukuba, Ibaraki, Japan). These cells secrete WFA-sialylated MUC1 and were thus used to examine the linear characteristics of the WFA-sialylated MUC1 sandwich ELISA. Values for all samples were calculated using the standard curve, and each value was calculated as a ratio relative to the standard curve. WFA-sialylated MUC1 values are expressed as µL of media/mL of serum (µL/mL). In each bile sample, all values were adjusted for the total protein concentration in bile (µg/mL) and expressed as nL of media/mL of bile/μg protein/mL of bile (nL/μg protein). The total protein concentration was measured using the Micro BCA protein assay reagent kit (Thermo Fisher Scientific, Fremont, CA, USA).

### Measurement of CA19-9 and CEA concentrations

Serum and bile concentrations of CA19-9 and CEA were measured using commercial CA19-9 and CEA ELISA kits (DRG Instruments GmbH, Marburg, Germany), respectively, following the manufacturer’s protocols. In each bile sample, all CA19-9 values were adjusted for the total protein concentration in bile (µg/mL) and expressed as U/μg protein.

### Statistical analysis

Data were analyzed using SPSS version 21.0 software (IBM Corp,, Armonk, NY, USA). The results are expressed as medians (ranges). To compare groups for all variables, one-way analysis of variance (ANOVA) was performed. Univariate analysis was performed on non-normally distributed data using nonparametric Mann–Whitney *U* tests. Categorical variables were compared using chi-square or Fisher’s exact tests, as appropriate. Receiver operating characteristic (ROC) curve analyses of WFA-sialylated MUC1, CA19-9, and CEA levels were performed to determine the optimal cut-off value for predicting the presence of biliary tract cancer. Results with *P* values of less than 0.05 were considered statistically significant.

## Results

### Serum and biliary levels of WFA-sialylated MUC1 in BTC/IhCC

In serum samples, WFA-sialylated MUC1 levels (µL/mL; median, range) were significantly higher in patients with either BTC or IhCC (340, 56–2000) and patients with perihilar CC (346, 131–1910), distal CC (252, 121–804), gallbladder carcinoma (325, 56–2000), and IhCC (498, 103–2000) than in control subjects (84, 0.6–230) and those with benign biliary tract diseases (124, 25–594; Table 1). Patients with IhCC showed higher WFA-sialylated MUC1 levels than those with tumors at other sites. Serum CA19-9 levels (U/mL) were significantly higher in patients with BTC or IhCC (74, 0.6–1314) and patients with perihilar CC (83, 1.4–998), gallbladder carcinoma (48, 0.8–1033), and IhCC (152, 0.8–1118) than in control subjects (9, 0.3–85) and those with benign biliary tract diseases (12, 0.2–1069; Table 1). Serum CEA levels (ng/mL) were significantly higher in patients with BTC or IhCC (2.6, 0.3–92.4) and patients with IhCC (3.2, 0.4–92.4) than in control subjects (1.3, 0.6–5.1) and patients with benign biliary tract diseases (2.6, 0.2–16.6; Table 1). When serum levels of WFA-sialylated MUC1, CA19-9, and CEA were analyzed in patients with BTC or IhCC, no correlation was observed between WFA-sialylated MUC1 and CA19-9 (*r* = 0.068, *n* = 303) or between WFA-sialylated MUC1 and CEA (*r* = 0.080, *n* = 303). However, there was a weak but significant positive correlation between CA19-9 and CEA (*r* = 0.221, *P* < 0.01, *n* = 303).

In bile samples, WFA-sialylated MUC1 levels (nL/µg protein; median, range) were significantly higher in patients with either BTC or IhCC (27, 10–653) and patients with perihilar CC (24, 10–653), distal CC (29, 10–533), gallbladder carcinoma (25, 11–341), and IhCC (60, 25–432) than in those with benign biliary tract diseases (7.4, 0.3–45; Table 2). Biliary CA19-9 levels (U/µg protein) were significantly higher in patients with BTC or IhCC (3372, 0.1–50156), perihilar CC (3468, 0.1–32149), and IhCC (4819, 102–50156) than in those with benign biliary tract diseases (1038, 0.1–11389; Table 2). When biliary levels of WFA-sialylated MUC1 and CA19-9 were analyzed in patients with BTC or IhCC, a weak but significant positive correlation was found between WFA-sialylated MUC1 and CA19-9 (*r* = 0.270, *P* < 0.01, *n* = 183).

**Table 2:** Cytology, WFA-sialylated MUC1 and CA19-9 levels in the bile samples of the study patients

Characteristics	Benign biliary disease (*n* = 115)	Total (*n* = 183)	Perihilar CC (*n* = 95)	Distal CC (*n* = 50)	Gallbladder carcinoma (*n* = 28)	Intrahepatic CC (*n* = 10)
Cytology, *n* (%)						
Negative	46 (40.0)	48 (26.2)	28 (29.5)	11 (22.0)	6 (21.4)	3 (30.0)
Positive suggestive	5 (4.3)	64 (35.0)	36 (37.9)	19 (38.0)	8 (28.6)	1 (10.0)
Positive	0 (0)	28 (15.3)	8 (8.4)	14 (28.0)	5 (17.9)	1 (10.0)
None	64 (55.7)	43 (23.5)	23 (24.2)	6 (12.0)	9 (32.1)	5 (50.0)
WFA-sialylated MUC1 (nL/µg protein)*	7.4 (0.3–45)	27 (10–653)^a^	24 (10–653)^a^	29 (10–533)^a^	25 (11–341)^a^	60 (25–432)^a^
CA19-9 (U/µg protein)*	1038 (0.1–11389)	3372 (0.1–50156)^a^	3468 (0.1–32149)^a^	2484 (3.5–48807)	3091 (48–17257)	4819 (102–50156)^a^

Values are expressed as medians (range)

Total represents the sum of cases with intrahepatic CC, perihilar CC, distal CC, and gallbladder carcinoma

*CC* cholangiocarcinoma, *WFA* Wisteria floribunda agglutinin, *MUC1* mucin core polypeptide 1, *CA19-9* carbohydrate antigen 19-9

*Adjusted by biliary protein concentration

^a^ significantly different from benign biliary disease

### Evaluation of the diagnostic capability of serum and biliary WFA-sialylated MUC1 levels

ROC curve analysis was performed to evaluate the diagnostic capability of serum WFA-sialylated MUC1 levels in discriminating patients with BTC/IhCC from control subjects and those with benign biliary tract diseases, and the results are shown in Table 3. The AUC values for WFA-sialylated MUC1, CA19-9, and CEA for differentiating patients with BTC/IhCC from control subjects were 0.963 (the cut-off value, 175.4 µL/mL), 0.857 (16.5 U/mL), and 0.767 (1.6 ng/mL), respectively (Table 3). The AUC values for differentiating patients with BTC/IhCC from those with benign biliary tract diseases were 0.873 (the cut-off value, 214.2 µL/mL), 0.753 (27.6 U/mL), and 0.523 (2.8 ng/ml), respectively (Table 3). In terms of diagnostic capability, the results suggest that serum levels of WFA-sialylated MUC1 may be a better serological biomarker than CA19-9 or CEA for distinguishing patients with BTC/IhCC from control subjects and those with benign biliary tract diseases.

**Table 3:** ROC curve analysis of the data on WFA-sialylated MUC1, CA19-9, and CEA levels in the serum and bile samples

Serum			Sensitivity	Specificity	AUC	Cut-off value
			%	%		
WFA-sialylated MUC1						µL/mL
Control	vs.	BTC/IhCC	89.8	88.6	0.963	175.4
Benign biliary disease	vs.	BTC/IhCC	77.6	78.0	0.873	214.2
CA19-9						U/mL
Control	vs.	BTC/IhCC	78.5	86.4	0.857	16.5
Benign biliary disease	vs.	BTC/IhCC	71.3	71.1	0.753	27.6
CEA						ng/mL
Control	vs.	BTC/IhCC	76.0	72.7	0.767	1.6
Benign biliary disease	vs.	BTC/IhCC	46.2	55.4	0.523	2.8
Bile			%	%		
WFA-sialylated MUC1						nL/µg protein
Benign biliary disease	vs.	BTC/IhCC	86.3	76.5	0.896	13.5
CA19-9						U/µg protein
Benign biliary disease	vs.	BTC/IhCC	68.9	60.0	0.690	1651

*WFA* Wisteria floribunda agglutinin, *MUC1* mucin core polypeptide 1, *CA19-9* carbohydrate antigen 19-9, *CEA* carcinoembryonic antigen, *BTC* biliary tract carcinoma, *IhCC* intrahepatic cholangiocarcinoma

Similarly, ROC curve analysis was performed to evaluate the diagnostic capability of biliary WFA-sialylated MUC1 levels in discriminating patients with BTC/IhCC from those with benign biliary tract diseases; the results are shown in Table 3. The AUC values for WFA-sialylated MUC1 and CA19-9 for differentiating patients with BTC/IhCC from those with benign biliary tract diseases were 0.896 (the cut-off value, 13.5 nL/µg protein) and 0.690 (1651 U/µg protein), respectively (Table 3). In terms of diagnostic capability, these results suggest that biliary levels of WFA-sialylated MUC1 may be a better biliary biomarker than levels of CA19-9 for distinguishing patients with BTC/IhCC from patients with benign biliary tract diseases.

**Fig. 1:**
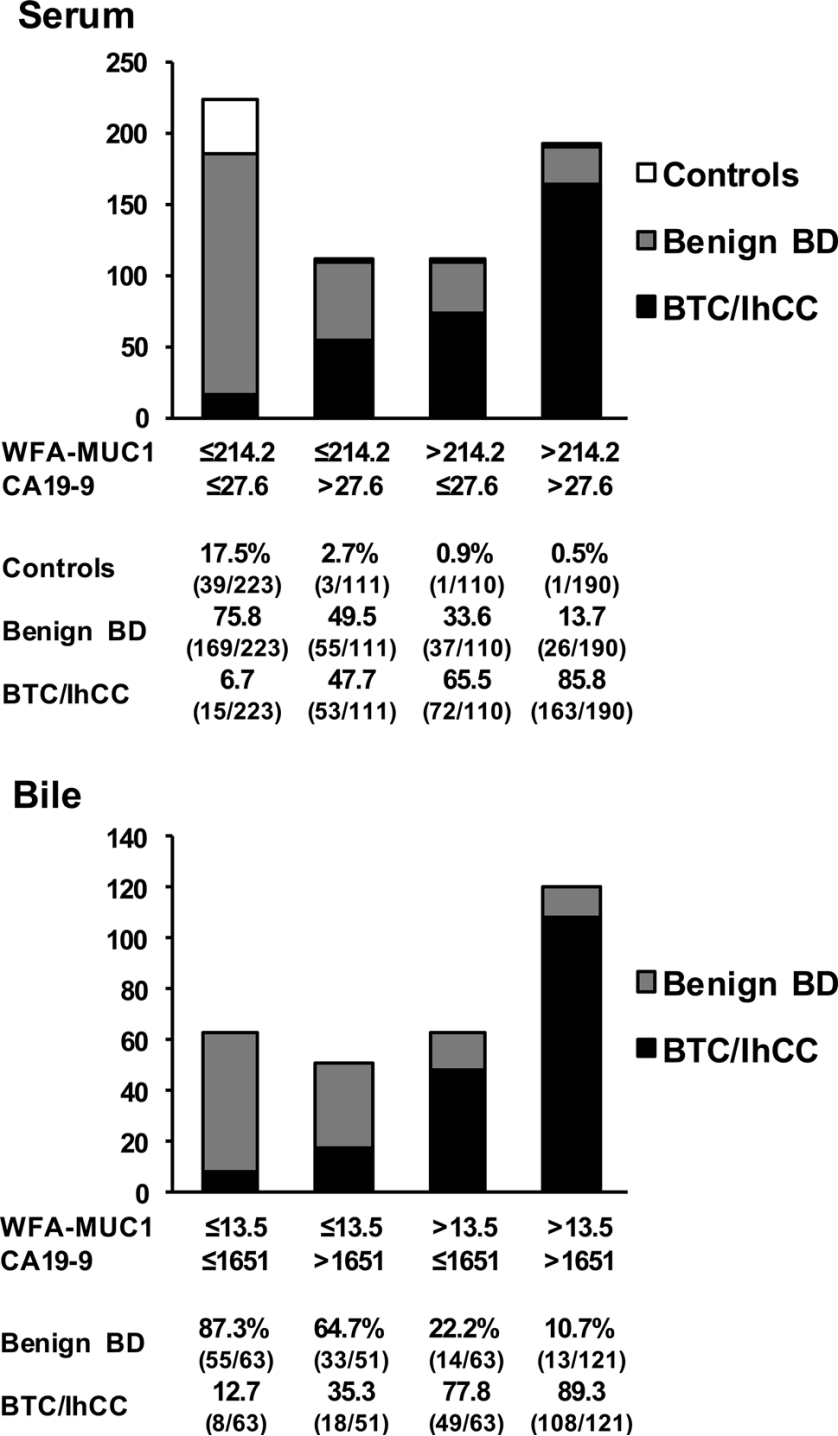
Subgroup analysis of total patients with either biliary tract carcinoma (*BTC*) or intrahepatic (*Ih*) cholangiocarcinoma (*CC*), patients with benign biliary tract disease (*BD*), and control subjects (*controls*) in terms of the positivity of WFA-sialylated MUC1 (*WFA*-*MUC1*) and CA19-9 in serum samples (*upper panel*) and bile samples (*lower panel*)

The diagnostic capability of the combined serum WFA-sialylated MUC1 and CA19-9 levels for discriminating patients with BTC/IhCC from patients with other conditions was determined using the following two cut-offs: WFA-sialylated MUC1 > 214.2 µL/mL and CA 19-9 > 27.6 U/mL. The results are shown in Fig. 1. Interestingly, 85.8 % of patients with WFA-sialylated MUC1 and CA19-9 above the cut-off values, 65.5 % of those with WFA-sialylated MUC1 above the cut-off value, 47.7 % of those with CA19-9 above the cut-off value, and 6.7 % of those with both markers below the cut-off values were found to have BTC or IhCC. In addition, 5.0 % of all patients with BTC or IhCC were those with both serum markers below the cut-off values. The diagnostic capability of the combined biliary WFA-sialylated MUC1 and CA19-9 levels in distinguishing patients with BTC/IhCC from those with benign biliary tract diseases was then determined using the following two cut-offs: WFA-sialylated MUC1 >13.5 nL/µg protein and CA 19-9 >1651 U/µg protein. The results showed that 89.3 % of patients with WFA-sialylated MUC1 and CA19-9 above the cut-off values, 77.8 % of those with WFA-sialylated MUC1 above the cut-off value, 35.3 % of those with CA19-9 above the cut-off value, and 12.7 % of those with both markers below the cut-off values had BTC or IhCC. In addition, 4.4 % of patients with BTC or IhCC were those with both biliary markers below the cut-off values. Thus, it is likely that a combination assay measuring WFA-sialylated MUC1 and CA19-9 levels in serum and bile samples would improve diagnostic accuracy for BTC/IhCC. Notably, the combined assay failed to detect a small proportion of patients with BTC/IhCC. Thus, for some patients, it may be difficult to exclude BTC/IhCC even when using a combined assay.

### Comparison of biliary cytology and WFA-sialylated MUC1 levels

**Fig. 2:**
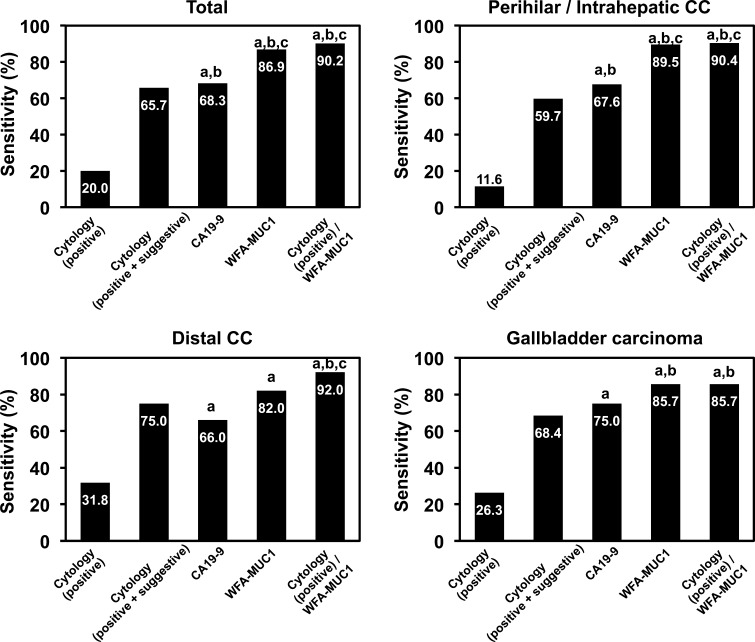
Comparison of the diagnostic power of biliary cytology, WFA-sialylated MUC1 (*WFA*-*MUC1*), and CA19-9 among all patients with biliary tract carcinoma or intrahepatic cholangiocarcinoma (*CC*), patients with perihilar CC or intrahepatic CC, those with distal CC, and those with gallbladder carcinoma

Biliary cytology was performed in 51 of the 115 patients with benign biliary tract diseases and 140 of the 183 patients with either BTC or IhCC (Table 2). In the group with benign biliary tract diseases, none of the 51 samples was classified as positive (class V), while five (9.8 %) were classified as suspected positive (class IIIb or IV). In these five samples, the WFA-sialylated MUC1 levels were found to be under the cut-off value (<13.5 nL/µg protein). In the group with BTC or IhCC, 48 of the 140 samples (34.3 %) were classified as negative (class I, II, or IIIa), 64 (45.7 %) were classified as suspected positive, and only 28 (20 %) were classified as positive. WFA-sialylated MUC1 levels were greater than the cut-off value (<13.5 nL/µg protein) in 85 % of the negative samples, 90 % of the suspected positive samples, and 94 % of the positive samples. Figure 2 shows the diagnostic sensitivity of cytology, CA19-9, and WFA-sialylated MUC1 in the bile samples. Sensitivity was 20.0 % for biliary cytology (positive), 65.7 % for cytology (positive plus suspected positive), 68.3 % for CA19-9 (cut-off value, 1651 U/µg protein), 86.9 % for WFA-sialylated MUC1 (cut-off value, 13.5 nL/µg protein), and 90.2 % for cytology (positive) plus WFA-sialylated MUC1. These results suggest that biliary WFA-sialylated MUC1 is a highly sensitive biomarker, and is superior to conventional biliary cytology and to the BTC tumor marker CA19-9 for the diagnosis of BTC/IhCC.

### Comparison of pathological findings and WFA-sialylated MUC11 levels

WFA-sialylated MUC1 levels in serum and bile samples from patients with either BTC or IhCC were compared with regard to pathological cancer stage and tumor tissue type. No significant difference was found in serum WFA-sialylated MUC1 levels based on stage and tumor tissue type (Table 4). Furthermore, WFA-sialylated MUC1 levels were similar between carcinomas at early and advanced stages and between well-differentiated and undifferentiated carcinomas. In contrast, serum levels of CA19-9 and CEA were significantly higher in stage IV carcinomas than in stage I or II carcinomas (Table 4). In addition, serum CA19-9 levels were significantly higher in moderately and poorly differentiated carcinomas than in papillary carcinomas (Table 4). Similarly, no significant difference in biliary WFA-sialylated MUC1 levels was found based on cancer stage or tumor tissue type (Table 4). Biliary CA19-9 levels also showed no significant difference according to cancer stage or tumor tissue type (Table 4).

**Table 4:** WFA-sialylated MUC1, CA19-9, and CEA levels in the serum and bile specimens of the study patients with respect to cancer pathological stages and histology

Serum sample	pStage					
	I	II	III	IV	I + II	III + IV
*n*	23	51	78	151	74	229
WFA-MUC1 (µL/mL)	313 (130–680)	397 (117–1411)	301 (56–1910)	377 (102–2000)	336 (117–1411)	346 (56–2000)
CA19-9 (U/mL)	15 (5–301)	33 (1–617)	76 (2–1314)	144 (1–1118)^a,b^	20 (1–617)	122 (1–1314)^c^
CEA (ng/mL)	1.7 (0.6–6.3)	1.9 (0.5–21)	2.8 (0.3–71)	2.7 (0.4–92.4)^b^	1.9 (0.5–21.0)	2.8 (0.3–92.4)^c^

Serum sample	Histology					
	Pap	Well	Mod	Por	Pap + Well	Mod + Por
*n*	25	79	157	42	104	199
WFA-MUC1 (µL/mL)	313 (130–483)	316 (117–1411)	358 (56–2000)	385 (112–2000)	314 (117–1411)	360 (56–2000)
CA19-9 (U/mL)	22 (5–311)	88 (1–949)	86 (1–1118)^a^	82 (1–1314)^a^	52 (1–949)	83 (1–1314)^c^
CEA (ng/mL)	1.9 (0.5–6.3)	2.4 (0.7–77.8)	2.7 (0.3–92.4)	2.4 (0.6–49.5)	2.4 (0.5–77.8)	2.7 (0.3–92.4)

Bile sample	pStage					
	I	II	III	IV	I + II	III + IV
*n*	16	30	52	85	46	137
WFA-MUC1 (nL/µg protein^d^)	26 (11–61)	30 (11–653)	28 (10–197)	25 (10–533)	27 (11–653)	27 (10–533)
CA19-9 (U/µg protein^d^)	3290 (102–25357)	2412 (0.1–11055)	4011 (37–48807)	3162 (47–50156)	2986 (0.1–25357)	3572 (37–50156)

Bile sample	Histology					
	Pap	Well	Mod	Por	Pap + Well	Mod + Por
*n*	17	45	92	29	62	121
WFA-MUC1 (nL/µg protein^d^)	39 (11–653)	22 (10–247)	29 (10–533)	23 (11–313)	27 (10–653)	26 (10–534)
CA19-9 (U/µg protein^d^)	4183 (102–25357)	3403 (37–48807)	3167 (0.1–20156)	3269 (45–16081)	3451 (37–48807)	3167 (1–50153)

Values are expressed as medians (range)

*Pap* papillary carcinoma, *Well* well-differentiated carcinoma, *Mod* moderately differentiated carcinoma, *Por* poorly differentiated carcinoma, *WFA* Wisteria floribunda agglutinin, *MUC1* mucin core polypeptide 1, *CA19-9* carbohydrate antigen 19-9, *CEA* carcinoembryonic antigen

*Adjusted by biliary protein concentration

^a^ Significantly different from pStage I

^b^ Different from pStage II

^c^ Different from pStages I + II

**Fig. 3:**
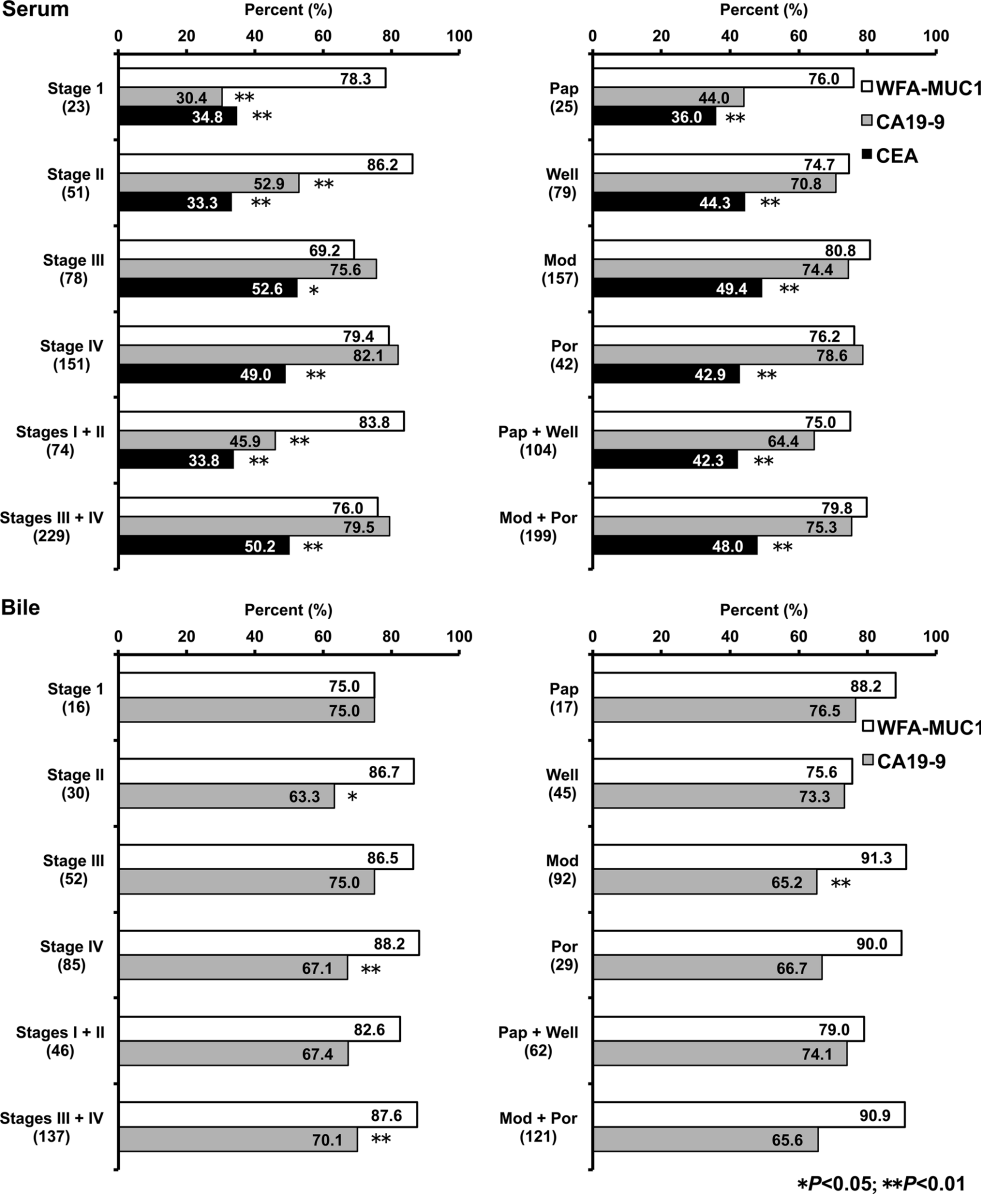
Pathological findings and positivity of WFA-sialylated MUC1 (*WFA*-*MUC1*), CA19-9, and CEA in serum samples (*upper panels*) and bile samples (*lower panels*) for all patients with either biliary tract carcinoma or intrahepatic cholangiocarcinoma

Figure 3 illustrates the diagnostic performance of WFA-sialylated MUC1 according to pathological cancer stage and tumor tissue type. To compare the diagnostic sensitivity of WFA-sialylated MUC1, CA19-9, and CEA, cut-off values were calculated as follows: WFA-sialylated MUC1 >214.2 µL/mL, CA19-9 >27.6 U/mL, and CEA >2.8 ng/mL for serum samples and WFA-sialylated MUC1 >13.5 nL/µg protein and CA19-9 >1651 U/µg protein for bile samples (Table 3). The results of serum sample analysis are shown in the upper panels of Fig. 3. WFA-sialylated MUC1 showed significantly higher diagnostic sensitivity than CA19-9 for stage I and II carcinomas, and was also found to be superior to CEA for stage I, II, III, and IV carcinomas. When samples were classified by tumor tissue type, WFA-sialylated MUC1 showed little difference in diagnostic sensitivity compared to CA19-9, but demonstrated significantly higher diagnostic sensitivity in comparison to CEA. The results of analysis of bile samples are shown in the lower panels of Fig. 3. For stage II and IV carcinomas, WFA-sialylated MUC1 showed significantly better diagnostic sensitivity than CA19-9. In contrast, WFA-sialylated MUC1 was superior to CA19-9 only for moderately differentiated carcinoma. When the results of bile samples were compared with those of serum samples, biliary WFA-sialylated MUC1 and CA19-9 showed similar diagnostic sensitivity in stage I and II carcinomas.

## Discussion

Several important findings emerged from this study. First, serum and biliary WFA-sialylated MUC1 levels were significantly higher in patients with either BTC or IhCC than in control subjects and patients with benign biliary tract diseases, with the highest levels observed in patients with IhCC. Second, serum and biliary WFA-sialylated MUC1 levels showed no significant difference in terms of pathological cancer stage or tumor tissue type. Third, in a comparison of diagnostic capability using ROC analysis, WFA-sialylated MUC1 was found to be superior to CA19-9 and CEA. In addition, the sensitivity of biliary WFA-sialylated MUC1 was superior to that of biliary cytology in diagnosing BTC/IhCC. Finally, diagnostic sensitivity for stage I and II carcinomas was significantly higher in serum WFA-sialylated MUC1 than in CA19-9 and CEA.

Although the variations in serum and biliary levels of WFA-sialylated MUC1 were larger within groups, the levels were significantly higher in patients with either BTC or IhCC and in patients with perihilar CC, distal CC, gallbladder carcinoma, and IhCC than in control subjects and those with benign biliary tract diseases (Tables 1 and 2). WFA-sialylated MUC1 levels were highest in the serum and bile from patients with IhCC . The background factors and biological mechanism underlying the difference between BTC/IhCC patients with low and high levels of WFA-sialylated MUC1 and the site-specific higher values in IhCC patients remain unknown. ROC analysis showed that WFA-sialylated MUC1 was superior to CA19-9 and CEA as a biomarker for BTC/IhCC. In addition, serum WFA-sialylated MUC1 showed superior diagnostic capability in discriminating patients with BTC/IhCC from healthy subjects and those with benign biliary tract diseases, whereas bile WFA-sialylated MUC1 was superior in distinguishing patients with BTC/IhCC from those with benign biliary tract diseases. Similar results were obtained in a previous validation study by Matsuda et al. using a small number of patients with BTC/IhCC [16].

Among all patients with BTC or IhCC, variations in the levels of CA19-9 were comparable to those of WFA-sialylated MUC1. However, our analysis of the correlation between WFA-sialylated MUC1 and CA19-9 in serum showed only a weak correlation, suggesting that WFA-sialylated MUC1 and CA19-9 could be used as biomarkers with different characteristics. Therefore, the application of a combination of WFA-sialylated MUC1 and CA19-9 in serum and bile for the detection of BTC/IhCC achieved improved diagnostic capability (Fig. 1). These results support the analysis by Matsuda et al. of serum samples from a small number of patients, in which measurement using a combination of WFA-sialylated MUC1 and CA19-9 was more accurate [16]. Therefore, such combined measurement may represent a superior diagnostic approach for the detection of BTC/IhCC in daily clinical practice.

Serum WFA-sialylated MUC1 levels in BTC/IhCC varied little by pathological cancer stage or tumor tissue type, with similar levels between early and advanced stages and between well-differentiated and undifferentiated carcinomas. In contrast, serum CA19-9 levels were significantly higher in stage IV carcinoma and in moderately and poorly differentiated carcinomas. These increased serum CA19-9 levels in advanced BTC/IhCC and in poorly differentiated carcinomas suggest that WFA-sialylated MUC1 could be applied as a biomarker with characteristics different from those of CA19-9.

There was no significant correlation between pathological malignancy (serosal invasion, lymph node metastasis, lymphatic vessel invasion, venous invasion, perineural invasion) and serum or biliary WFA-sialylated MUC1 levels (data not shown). The diagnostic sensitivity of serum WFA-sialylated MUC1 in stage I and II carcinomas was excellent, and was highly superior to that of CA19-9 and CEA. This finding has significant clinical implications, given that early diagnosis of BTC/IhCC with conventional tumor markers is limited by several factors. Earlier detection of BTC/IhCC can significantly improve prognosis.

In the clinical setting, it is often difficult to determine whether biliary stenosis is caused by a benign or malignant lesion [20, 21]. Despite the use of advanced imaging techniques, diagnosis is uncertain [22]. Moreover, studies have found that 3–17 % of patients with suspected malignant stenosis were eventually diagnosed with a benign lesion through postoperative histological examination [23, 24]. In many cases, diagnosis is based on bile cytology. However, the positive rate of biliary cytology is low [25]. Indeed, in our study, this rate was only 20 %, underscoring the difficulty in distinguishing BTC/IhCC from benign biliary tract diseases using biliary cytology. In the validation study performed by Matsuda et al. [15], despite the small sample size (30 patients with BTC and 38 patients with benign biliary tract diseases), biliary WFA-sialylated MUC1 levels were shown to definitively distinguish BTC/IhCC from benign biliary tract diseases (sensitivity = 90.0 %, specificity = 76.3 %, area under the curve [AUC] = 0.85). In our study, with a larger sample size of 183 patients with BTC or IhCC and 115 patients with benign biliary tract diseases, ROC analysis showed results similar to those reported by Matsuda et al. (Table 2; sensitivity = 86.3 %, specificity = 76.5 %, AUC = 0.896).

Based on the cut-off value obtained in this study, biliary WFA-sialylated MUC1 levels showed significantly superior diagnostic sensitivity for BTC/IhCC compared with biliary cytodiagnosis, using the same sample (Fig. 2; 20.0 vs. 86.9 %). A combination of biliary cytodiagnosis and WFA-sialylated MUC1 levels yielded diagnostic sensitivity of 90.2 %. Thus, the combination of cytodiagnosis and WFA-sialylated MUC1 improved diagnostic accuracy in the clinical setting.

In summary, this clinical study demonstrated that WFA-sialylated MUC1 levels in serum or bile samples were useful as biomarkers to distinguish patients with BTC/IhCC from control subjects and those with benign biliary tract diseases. The measurement of WFA-sialylated MUC1 levels may dramatically improve diagnostic accuracy for BTC/IhCC in routine clinical care. The incidence of BTC/IhCC has continued to increase, and chronic biliary tract diseases are proven risk factors for the development and progression of IhCC. However, in recent large-scale epidemiological surveys, lifestyle-related diseases such as obesity and diabetes mellitus were implicated as well [25, 26]. Therefore, the planning and provision of appropriate health care is important in efforts to reverse the rising incidence of BTC/IhCC.

## List of abbreviations

BTC: Biliary tract carcinoma
CA19-9: Carbohydrate antigen 19-9
CEA: Carcinoembryonic antigen
CC: Cholangiocarcinoma
Ih: Intrahepatic
ROC: Receiver operating characteristic
WFA: *Wisteria floribunda* agglutinin
